# Adherence and Patients' Experiences with the Use of Capecitabine in Daily Practice

**DOI:** 10.3389/fphar.2016.00310

**Published:** 2016-09-21

**Authors:** Lonneke Timmers, Christel C. L. M. Boons, Dirk Mangnus, Peter M. Van de Ven, Pieter H. Van den Berg, Aart Beeker, Eleonora L. Swart, Richard J. Honeywell, Godefridus J. Peters, Epie Boven, Jacqueline G. Hugtenburg

**Affiliations:** ^1^Department of Clinical Pharmacology and Pharmacy, VU University Medical CenterAmsterdam, Netherlands; ^2^Department of Pharmacy, Slingeland ZiekenhuisDoetinchem, Netherlands; ^3^Department of Epidemiology and Biostatistics, VU University Medical CenterAmsterdam, Netherlands; ^4^Department of Internal Medicine, Tergooi HospitalHilversum, Netherlands; ^5^Department of Internal Medicine, Spaarne HospitalHoofddorp, Netherlands; ^6^Department of Medical Oncology, VU University Medical CenterAmsterdam, Netherlands; ^7^The EMGO Institute for Health and Care Research, VU University Medical CenterAmsterdam, Netherlands

**Keywords:** capecitabine, medication adherence, patient-reported symptoms, dose adjustments, pharmacokinetics, oral anticancer agent

## Abstract

**Introduction:** Capecitabine is a widely prescribed oral anticancer agent. We studied medication adherence and explored its use in daily practice from a patients' perspective.

**Patients and Methods:** Patients (*n* = 92) starting capecitabine were followed up to five 3-week cycles. Adherence was assessed using a pill count, pharmacy data and dosing information from the patients' medical file. Self-reported adherence was measured using the Medication Adherence Report Scale (MARS). At baseline and during week 2 of cycles 1, 3, and 5, patients filled out questionnaires about quality of life, symptoms, attitude toward medicines and disease and use in daily practice. Simultaneously, blood samples were taken to determine the area under the curve (AUC) of 5′-deoxy-5-fluorouridine (5′-DFUR), 5-fluorouracil (5-FU), and α-fluoro-β-alanine (FBAL) by a population pharmacokinetic model. Associations between AUCs and patient-reported symptoms were tested for cycles 3 and 5.

**Results:** Most patients (84/92; 91%) had an adherence rate of ≥95 and ≤ 105%. The percentage of patients reporting any non-adherence behavior measured with MARS increased from 16% at cycle 1 to 29% at cycle 5. Symptoms were reported frequently and the dosing regimen was adjusted by the physician at least once in 62% of patients. In multivariate analysis the probability of an adjustment increased with the number of co-medication (OR 1.19, 95% CI: 1.03–1.39) and a stronger emotional response to the disease (OR 1.32, 95% CI: 1.10–1.59). The AUC of 5′-DFUR was associated with weight loss (OR 1.10, 95% CI: 1.01–1.19), AUC of FBAL with hand-foot syndrome (OR 0.90, 95% CI: 0.83–0.99), rhinorrhea (OR 1.21, 95% CI: 1.03–1.42 weight loss (OR 1.09, 95% CI: 1.00–1.20) and depression (OR 0.90, 95% CI: 0.82–0.99). Side effects were reported by one third of patients as the reason to discontinue treatment.

**Conclusion:** Adherence to capecitabine was generally high. Nevertheless, adherence measured with MARS decreased over time Adherence management to support implementation of correct capecitabine use is specifically relevant in longer term treatment. In addition, it appears that adverse event management is important to support persistence. With the extending armamentarium of oral targeted anticancer agents and prolonged treatment duration, we expect the issue of medication adherence of increasing importance in oncology.

## Introduction

Capecitabine is registered for treatment of patients following surgery of stage III (Dukes' stage C) colon cancer in the adjuvant setting, for treatment of metastatic colorectal cancer, first-line treatment of advanced gastric cancer and for treatment of locally advanced or metastatic breast cancer (European Medicines Agency (EMEA), 2015). Capecitabine is an oral prodrug of 5-fluorouracil (5-FU). Oral administration is more convenient and most patients prefer oral medication provided similar efficacy (Liu et al., 1997; Borner et al., 2001). 5-FU has largely been replaced by capecitabine, since the latter drug is considered equally effective (Cassidy et al., 2002; Van Cutsem et al., 2004; Twelves et al., 2005), has a generally better toxicity profile apart from hand-foot syndrome (HFS) (Cassidy et al., 2002; Twelves et al., 2005) and is more cost-effective (Cassidy et al., 2006; Ward et al., 2006). Capecitabine is commonly prescribed in a 3-week treatment cycle, with doses on days 1 through 14, followed by a 7-day rest period. The dosing regimen is even more complex as the capsules should be used twice daily within 30 min after a meal with a 12-h interval. Furthermore, to obtain the required dosage patients need to take several capsules (e.g., when prescribed a daily dose of 2300 mg, a patient has to take 2 capsules of 500 mg and 1 capsule of 150 mg in the morning and in the evening; European Medicines Agency (EMEA), 2015). The starting dose depends on previous treatments, combination with other cytotoxic agents, renal function, and knowledge of dihydropyrimidine dehydrogenase (DPD) deficiency and is calculated on the basis of the body surface area (BSA). Dose reduction and/or delay are guided by the occurrence of toxicity.

As a consequence of oral administration of anticancer agents responsibility of intake has shifted toward patients and non-adherence is an issue to deal with. Adherence to capecitabine, appears to be higher (Macintosh et al., 2007; Mayer et al., 2009; Regnier Denois et al., 2011; Simons et al., 2011; Winterhalder et al., 2011; Bhattacharya et al., 2012; Krolop et al., 2013; Patel et al., 2013; Thivat et al., 2013; Walter et al., 2013; Timmers et al., 2014; Zahrina et al., 2014; De Figueiredo and Forones, 2014) than adherence to long-term medication in chronic disease (Sabaté, 2003; Osterberg and Blaschke, 2005). Assessment of adherence to capecitabine treatment is challenging because of the dosing regimen which is complex and the frequent necessity of individual adjustments. For assessment of medication adherence it is essential to use a method to distinguish patients' non-adherence from physicians' adjustments during treatment.

Several factors can be related to non-adherence (Verbrugghe et al., 2013; Mathes et al., 2014). Toxicity from drugs is an important factor in non-adherence (Verbrugghe et al., 2013). Patient-reported symptoms are a better reflection of the subjective daily health status than physician's scoring systems (Basch et al., 2009). When adverse events are assessed in relation with adherence, it is consistent to study them from a patients' point of view as patient-reported symptoms might have a stronger relationship with patients' non-adherence.

According to Leventhal's common sense model (CSM) patients' perception of and beliefs about their illness are important factors in their reactions and behavior to health threats (McAndrew et al., 2008). Beliefs about medicines are also associated with medication adherence (Horne et al., 2013) in various diseases.

Pharmacokinetic behavior of capecitabine is complex and there is considerable variability in metabolite concentrations among patients caused by multiple factors (Reigner et al., 2001). Gieschke et al. (2003) have demonstrated a relation between systemic exposure to the capecitabine metabolites 5′-DFUR, 5-FU, and FBAL and efficacy or toxicity. Yen-Revollo et al. (2008) have given mounting evidence that HFS seems to be caused by products of DPD-initiated catabolic degradation of 5-FU. With the use of capecitabine in daily practice, drug exposure might be a factor of influence on medication adherence.

The present study was designed to to get more insight into patients' experiences with the use of capecitabine in daily practice and the various aspects that govern adherence. To that end, we studied medication adherence in patients with cancer starting the use of capecitabine and the influence of patients' attitudes and side-effects on adherence. Furthermore, we explored possible relationships between patient characteristics, disease characteristics, symptoms, quality of life, patients' beliefs and attitude toward disease and medicines, dose adjustments, reasons for discontinuation and exposure to 5′-DFUR, 5-FU, and FBAL and adherence.

## Patients and methods

### Study design

In this prospective observational cohort study (Timmers et al., 2012), conducted between June 2010 and January 2012 in 10 Dutch hospitals, patients who started capecitabine treatment were followed up to five 3-week treatment cycles. The study was approved by the Medical Ethics review board of VU University Medical Center (VUMC, Amsterdam, the Netherlands), as well as the boards of each participating center. Written informed consent was obtained from all patients. The study was registered in the Netherland Trial Register under number NTR2324 and the protocol was published (Timmers et al., 2012).

### Patients

Regardless of the indication, patients starting treatment with capecitabine were eligible for participation. Exclusion criteria were: age younger than 18 years and inability to fill out a Dutch questionnaire.

### Data collection

#### Disease characteristics and dose adjustments

Information on indication for treatment with capecitabine, BSA, the starting dose, dose adjustments, and concomitant cytotoxic agents was retrieved from the patient's medical file. Adjustments were categorized as: dose reduction (lowering the daily dose), shortening of the period of use (less than 14 consecutive days of capecitabine at the beginning of the 3-week cycle) and delay (the next cycle starting more than 21 days after the start of the previous cycle).

#### Medication adherence

**Figure 1:**
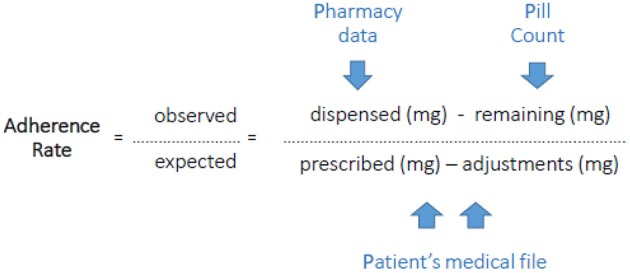
**PPP-method (Pill count-Pharmacy records-Patients' file) to measure the Adherence Rate**.

The overall adherence rate in the implementation phase was assessed during the studied period with a pill count method which corrects for the adjustments made by the physician. We refer to this as the PPP-method (Pill count-Pharmacy records-Patients' file). Patients were contacted unannounced by phone after finishing the fifth cycle or earlier when they prematurely discontinued. They were asked to count their remaining pills and were whether they had returned pills to the pharmacy or disposed pills in any other way. Dispensing records of the pharmacy (or pharmacies) and the initially prescribed number of pills as well as all adjustments during use retrieved from the patient's medical file were collected. This information was plotted in a calendar during the studied period (form start till the moment of pill count). Adherence was expressed as the percentage of the total amount in mg the patient used in the studied period toward the total amount in mg the patient was supposed to use in the studied period. This was calculated by dividing the number of pills dispensed minus the pill count by the initially prescribed capecitabine dosing regimen minus adjustments of the dosing regimen (Figure 1). In addition, medication adherence behavior was assessed by a patient-reported questionnaire (Medication Adherence Report Scale, MARS; Horne et al., 2001). Furthermore, the blood samples were used to get insight into medication adherence. When capecitabine nor one of its primary metabolites (5′-DFCR, 5′-DFUR, 5-FU) could be detected in the period 20 min–12 h after the moment of intake that was reported by the patient, the patient was assumed to be non-adherent at that time-point.

#### Patient-reported questionnaires

Patients filled out a questionnaire at baseline and during treatment, in the second week of cycles 1, 3, and 5. including education, living status, occupation, all co-medication, quality of life (SF-12) (Gandek et al., 1998), illness perceptions (Brief IPQ) (Broadbent et al., 2006), patient's beliefs about medicine in general and toward capecitabine (BMQ) (Horne et al., 1999). adherence (MARS) (Horne et al., 2001), food intake in relation to capecitabine intake and type and grade of symptoms. Symptoms occurring in ≥1% of patients according to the European Public Assessment Report (EPAR) of capecitabine (European Medicines Agency (EMEA), 2015) were included in the questionnaire. Answers could be scored on a 5 point Likert scale (not at all, a little bit, rather, a lot, very much). Symptoms scored as “a lot” or “very much” were considered as “severe.” When a patient discontinued capecitabine treatment, the reasons for discontinuation were enquired.

#### Exposure to metabolites

A blood sample was collected in the second week of cycles 1, 3, and 5. The time of blood withdrawal and the time of the last capecitabine intake were recorded. At each withdrawal patient's length and weight were documented. Blood samples were analyzed by validated liquid chromatography/tandem mass spectrometry methods for plasma concentrations of capecitabine, 5′-DFCR, 5′-DFUR, 5-FU and FBAL (Deenen et al., 2013). Alkaline phosphatase (AF) and creatinine concentrations were analyzed from the same blood sample. Creatinine clearance was calculated using the Cockcroft-Gault formula (Cockcroft and Gault, 1976). For *post-hoc* estimation of the area under the curve (AUC) of the metabolites, the data were incorporated in the NONMEM program (version VI), using a population pharmacokinetic model described by Gieschke et al. (2003). Apart from the concentration of the metabolites 5′-DFUR, 5-FU, and FBAL, the model required capecitabine dose, time between intake of capecitabine and blood sample collection, BSA, AF concentration and creatinine clearance to estimate the AUC. Samples collected within 20 min after intake, those with an unknown time-point of last capecitabine intake and undetectable plasma levels of 5′-DFUR or 5-FU were excluded from further analyses.

### Statistical methods

The baseline descriptive data were presented as frequencies (percentages) for categorical variables and as the means with standard error or median with range for continuous data. The associations between BMQ-subscales, Brief IPQ-subscales and symptoms and a MARS <25-score at cycle 3, were analyzed univariate by means of logistic regression with MARS <25-score as the dependent variable. Associations between symptoms at cycle 1 and a decreased dose at the start of cycle 2 was tested using the X^2^-test or Fisher's exact test when appropriate. Associations between reporting of symptoms and any adjustment in dosing regimen (shortening of period of use of cycle 1, delaying cycle 2 and/or adjusting during cycle 1 or at the start of cycle 2) were tested in a similar way. The influence of baseline characteristics as factors on dose reduction at the start of any new cycle, and any adjustments in dosing regimen during the study period, respectively, was tested using univariate logistic regression. Factors tested significantly in univariate analysis (*p* < 0.05) were included in a multivariate logistic regression using forward selection. To test the relationship between the AUC of a metabolite of capecitabine and a patient-reported symptom at cycles 3 and 5, a Generalized Estimating Equations (GEE) model was fitted with presence of symptoms as the dependent variable and the AUC measured at the same cycle as the independent variable. GEE was used to take into account within-patient correlation between the dependent variable at cycles 3 and 5. For all analyses, a two-tailed significance level of 0.05 was used. *P*-values below this level were considered statistically significant. The statistical analyses were performed with SPSS 20.0 for Windows (IBM Corp, Armonk, NY, USA).

## Results

### Baseline characteristics

A total of 92 patients were included, of whom 87, 70, and 57 were still on treatment in the second week of cycles 1, 3, and 5, respectively. Supplementary Table S1 provides information on the number of patients for whom data were available at each time-point. All patients used capecitabine in the usual 3-week cycle regimen. Baseline characteristics of the patients are listed in Table 1. Patients (62% male, median age 64.4 years) had most frequently a diagnosis of colorectal cancer (66/72%), 16 had breast cancer (17%), four had pancreatic cancer (4%), and six had another type of cancer (oesophageal, head-and-neck, and unknown origin; 7%).

**Table 1:** **Baseline characteristics**.

Age (yr) median	64.4
Range	39.2–86.8
Sex (N (%))	
Male	57 (62.0)
Female	35 (38.0)
Education (N (%))	
Low level	25 (27.2)
Higher level	67 (72.8)
Living status (N (%))^a^	
Living alone	16 (17.6)
Living together	75 (83.4)
Occupation (N (%))^b^	
Paid work	25 (28.4)
No paid work	63 (71.6)
Co-medication^c^	
% of patients with co-medication	78.9
Number of medicines, median	2
Number of medicines, range	0–14
Initial capecitabine dose	
Median (mg/m^2^/d)	1975
Range (mg/m^2^/d)	1046–2609
Median (mg/d)	4000
Range (mg/d)	2000–5300
Type of cancer (N (%))	
Colorectal cancer	66 (71.7)
- Adjuvant	17 (18.5)
- Metastatic	49 (53.3)
Breast cancer	16 (17.4)
Gastric cancer	4 (4.3)
Other	6 (6.5)
Capecitabine regimen (N (%))	
Single agent	27 (29.3)
Combination	65 (70.7)

*Abbreviations: yr, years; mg/d, milligrams per day; mg/m^2^/d, milligrams, per square meters per day*.

a *One missing*.

b *Four missings*.

c *Two missings*.

### Treatment and adjustments to the dosing regimen

The median starting dose of capecitabine was twice daily 2000 mg (range: twice daily 1000–2650 mg a day), corresponding with a median dosing regimen of twice daily 988 mg/m^2^/d (range 0523–1305). The initial dosing regimen was adjusted at least once in 62% of the patients. This could either be a dose reduction (30%), dose increase (13%), shortening of the period of use (21%), or delay (35%). In The type, frequency and time-points of dose adjustments are described in detail in Supplementary Table S2.

### Medication adherence

The adherence rate (implementation phase) could be calculated for 67 patients (73%). For the remaining 27%, either the pill count was missing or data from the pharmacy or medical file were incomplete. Within the studied period the mean and median adherence rate were 99.3 and 100.0%, respectively. Most patients (91%) had an adherence rate of ≥95– ≤ 105%. In five patients (7%) the adherence rate was <95% and in two of them (3%) this was <90%. One patient had an adherence rate >105%.

Self-reported adherence measured with MARS is presented in Table 2. Non-adherence increased over time. The percentage of patients reporting non-adherence on any of the five MARS statements was 16% at cycle 1, 23% at cycle 3, and 29% at cycle 5. The most common statement was “forgetting to take it” (12, 20, and 26% at respectively cycles 1, 3, and 5). Over-adherence was mentioned by one patient at cycle 5.

**Table 2:** **Use of capecitabine, quality of life, and patients' beliefs**.

	**T0**	**Cycle 1^a^**	**Cycle 3^a^**	**Cycle 5^a^**
	***N* = 92**	***N* = 77**	***N* = 66**	***N* = 56**
**ADHERENCE (MARS)**				
MARS < 25 score (%)	n.a.	15.7	23.1	28.8
I forget to take it (%)	n.a.	12.2	20.0	25.5
I alter the dose (%)	n.a.	2.8	6.2	9.3
I stop taking it for a while (%)	n.a.	4.3	1.5	5.6
I decide to miss out a dose (%)	n.a.	4.2	3.1	9.4
I take less than instructed (%)	n.a.	1.4	1.5	3.8
**OVERUSE**				
I use more than prescribed (%)	n.a.	0.0	0.0	1.9
Reminder method (%)	n.a.	61.5	66.2	73.2
Incorrect timing of intake (%)	n.a.	13.2	32.8	24.1
Intake with grapefruit (%)	n.a.	0.0	1.6	3.6
**SF-12 (MEAN ± SD)**				
Physical component (0–100)	37.1 ± 9.3	38.4 ± 9.9	37.7 ± 9.9	37.4 ± 11.1
Mental component (0–100)	47.1 ± 7.5	48.5 ± 6.7	49.0 ± 5.8	50.4 ± 5.4
**BMQ (MEAN ± SD)**				
General overuse (4–20)	10.4 ± 2.4	10.5 ± 2.9	10.6 ± 2.6	10.6 ± 2.3
General harm (4–20)	9.7 ± 2.52	10.1 ± 2.5	9.5 ± 2.5	9.8 ± 2.5
Specific necessity (5–25)	19.5 ± 3.3	19.5 ± 3.6	19.2 ± 3.9	18.6 ± 4.1
Specific concern (5–25)	14.3 ± 4.0	14.2 ± 3.9	14.0 ± 4.4	14.1 ± 3.6
Nec-conc differential (−20–20)	5.1 ± 5.3	5.1 ± 4.6	5.2 ± 5.2	4.6 ± 5.5
**BRIEF IPQ (MEAN ± SD)**				
Consequences (0–10)	6.8 ± 2.6	6.8 ± 2.8	6.8 ± 2.5	6.6 ± 2.4
Time line (0–10)	7.4 ± 2.8	7.5 ± 2.6	7.4 ± 2.9	7.1 ± 3.1
Personal control (0–10)	4.6 ± 2.9	4.5 ± 2.6	4.5 ± 3.0	4.3 ± 3.0
Treatment control (0–10)	7.8 ± 1.8	7.4 ± 2.1	7.8 ± 1.8	8.0 ± 1.6
Identity (0–10)	5.1 ± 2.9	5.0 ± 2.8	5.8 ± 3.0	5.5 ± 2.9
Concern (0–10)	7.3 ± 2.4	7.6 ± 2.3	6.8 ± 2.8	6.8 ± 2.4
Understanding (0–10)	6.1 ± 2.9	5.9 ± 3.1	5.9 ± 3.1	6.1 ± 3.0
Emotional response (0–10)	4.9 ± 2.8	4.8 ± 2.9	4.7 ± 2.5	4.5 ± 2.6

*Abbreviations: sd, standard deviation; MARS, Medication Adherence Report Scale; SF-12, SF-12 Health Survey; BMQ, Beliefs about Medicines Scale; Brief IPQ, Brief Illness Perception Questionnaire*.

a *Measured in week 2 of cycle. Missings excluded in frequency analyses*.

There were three blood samples (1.9%) without any detectable concentrations of capecitabine, 5′-DFCR, 5′-DFUR, and 5-FU, while the patient stated to have taken the last capecitabine within the period 12 h–20 min prior to blood collection. These samples belonged to three different patients (two at cycle 3 and one at cycle 5).

The majority of patients (>60%) reported to use a reminder method to support the capecitabine intake. At cycles 1, 3, and 5, respectively 13, 33, and 24% of patients did not always time their capecitabine with the intake of food as recommended (Table 2).

At the end of the observation period, 35 patients had discontinued treatment. Of these patients, 16 filled out the questions about discontinuation: 7 (44%) reported side-effects as the reason to stop treatment with capecitabine, 4 (25%) discontinued because of lack of clinical efficacy, the remaining for other or unknown reasons. For 11 (69%) of these 16 patients, physicians indicated the same reason to discontinue. Physicians reported progression in 8 (50%) of patients and adverse events in 4 (25%). Most patients (82%) indicated that treatment was discontinued on the initiative of their physician.

### Patients' beliefs, attitude, and quality of life

Table 3 shows the mean scores of items on SF-12, BMQ, and Brief IPQ at baseline, as well as in week 2 of cycles 1, 3, and 5. The BMQ classification into four attitudinal groups based on the patients' responses at baseline was: 50.0% accepting (high necessity, low concerns), 44.0% ambivalent (high necessity, high concerns), 3.6% indifferent (low necessity, low concerns), and 2.4% skeptical (low necessity, high concerns).

**Table 3:** **Patient-reported symptoms**.

	**% patients with any symptom**				% patients with severe^*^ **symptom**			
	**T0**	**Cycle 1^a^**	**Cycle 3^a^**	**Cycle 5^a^**	**T0**	**Cycle 1^a^**	**Cycle 3^a^**	**Cycle 5^a^**
*N*	92	77	66	56	92	77	66	56
Hand-foot syndrome	26.4	64.4	76.9	94.5	6.9	6.8	33.8	52.7
Fatigue	78.7	83.1	87.7	91.1	0	27.3	0	0
Flatulence	42.5	61.3	65.6	67.9	2.3	10.7	14.1	13.2
Change of taste	22.7	53.9	54.7	65.5	2.3	9.2	9.4	12.7
Skin problems	11.4	27.6	38.5	61.8	0	2.6	7.7	12.7
Dry mouth	34.1	44.0	52.3	61.8	0	6.7	7.7	10.9
Nausea	20.9	49.3	45.2	54.5	2.3	9.6	6.5	7.3
Breathing problems	33.7	40.3	46.2	53.7	2.3	2.6	6.2	7.4
Stomach ache	33.0	35.5	42.2	51.9	4.5	9.2	6.3	11.1
Rhinorrhea	27.3	32.9	33.8	50.9	0	3.9	4.6	1.9
Muscular pain	44.9	43.4	51.5	50.9	7.9	10.5	13.6	9.4
Loss of appetite	43.3	51.9	40.6	48.1	6.7	15.6	9.4	7.4
Diarrhea	21.8	41.9	35.5	43.6	0	2.7	4.8	7.3
Nail problems	7.1	8.0	23.8	43.6	1.2	0	3.2	7.3
Eye problems	10.2	18.4	39.1	43.4	0	1.3	4.7	11.3
Dizziness	16.1	30.7	37.5	40.7	1.1	0	4.7	5.6
Headache	16.1	33.8	34.9	37.0	1.1	4.1	3.2	7.4
Insomnia	40.9	32.9	41.5	37.0	3.4	9.2	6.2	3.7
Constipation	38.8	36.0	36.5	36.4	5.9	5.3	9.5	7.3
Depression	40.2	38.2	38.5	36.4	4.6	6.6	0	1.8
Weight loss	39.8	47.4	47.6	29.6	2.3	11.8	7.9	3.7
Alopecia	4.6	10.5	27.7	26.4	2.3	3.9	4.6	5.7
Mucositis	5.7	13.3	12.7	25.5	0	1.3	1.6	1.8
Fever	12.6	16.0	17.2	16.7	0	4.0	4.7	0
Oedema	9.2	9.3	23.1	16.4	1.1	1.3	4.6	3.6

*Symptoms presented in order of prevalence during cycle 5*.

* All symptoms scored as “a lot” and “very much” were considered “severe.”

a *Symptoms measured in week 2 of cycle. Missings excluded in frequency analyses*.

### Patient-reported symptoms

Patient-reported symptoms at baseline and in week 2 of cycles 1, 3, and 5 are listed in Table 3. The majority of patients (79%) reported any fatigue at baseline, which was severe in 27% of patients in cycle 1 only. HFS was the most severe symptom during treatment of which the prevalence substantially increased over time. Any HFS and severe HFS increased from, respectively, 26 and 7% at baseline to 95 and 53% of patients in cycle 5.

### Exposure to metabolites

Baseline characteristics required for the pharmacokinetic analyses (length, weight, BSA, creatinine, creatinine clearance, AF, and capecitabine doses) are listed in Table 4. A total of 160 blood samples were collected. Three samples were excluded because they were collected within 20 min after intake, and three samples because of an unknown time-point of the last capecitabine intake. In three samples not any detectable metabolite was measured. Furthermore, 15 samples were collected more than 12 h after the last capecitabine intake. The median capecitabine dose was 995, 997, and 988 mg/m^2^ twice daily at cycle 1, 3, and 5, respectively. The median AUCs (mg*h/L) in cycle 1 of 5′-DFUR, 5-FU, and FBAL were 14.4, 0.86, 22.6, respectively.

**Table 4:** **Characteristics pharmacokinetics**.

**All samples**		**Cycle 1^a^**	**Cycle 3^a^**	**Cycle 5^a^**
		***N* = 58**	***N* = 41**	***N* = 38**
Length (cm)	Median	172	173	172
	Range	156–195	156–196	156–196
Weight (kg)	Median	77.5	77.9	77.0
	Range	51–130	50–112	49.5–124
BSA (m^2^)	Median	1.90	1.87	1.87
	Range	1.49–2.44	1.49–2.44	1.47–2.40
Creatinine (μmol/l)	Median	77.5	74	78.5
	Range	51–146	52–289	36–150
Creatinine clearance (ml/min)	Median	95.1	100.0	95.7
	Range	48.4–184.0	20.3–156.3	45.9–194.5
ALP (U/l)	Median	82.5	73.0	84.0
	Range	38–373	42–779	39–297
Dose capecitabine (mg/2dd)	Median	2000	2000	1800
	Range	1000–2650	1150–2500	1000–2250
Dosing regimen (mg/m^2^/2dd)	Median	996	997	975
	Range	524–1305	605–1278	539–1093
AUC 5′-DFUR (mg^*^ h/L)^b^	Mean ± sd	14.39 ± 3.10	13.85 ± 4.02	12.68 ± 4.03
	Median	14.39	13.33	12.47
	Range	6.97–22.28	7.45–26.26	5.03–26.16
AUC 5-FU (mg^*^ h/L)*^b^*	Mean ± sd	0.953 ± 0.449	1.043 ± 0.448	1.180 ± 0.314
	Median	0.860	0.936	1.106
	Range	0.368–2.697	0.447–2.228	0.314–3.632
AUC FBAL (mg^*^ h/L)*^b^*	Mean ± sd	22.71 ± 4.28	21.23 ± 3.59	20.18 ± 4.18
	Median	22.63	21.37	20.78
	Range	13.48–31.94	15.56–30.12	11.46–28.64

*Abbreviations: sd, standard deviation; BSA, body surface area; ALP, alkaline phosphatase; U, units; 2dd, twice daily; AUC, area under the curve; 5′-DFUR = 5′-deoxy-5-fluorouridine; 5-FU, 5-fluorouracil; FBAL, α-fluoro-β-alanine*.

a *In week 2 of cycle*.

b *Undetectable plasma concentrations were excluded*.

### Factors associated with non-adherence (implementation phase)

As only six patients (9%) had an adherence rate of ≤ 95% or >105% (measured with the pill count) no factors, such as patients' beliefs and attitude, patient-reported symptoms, or exposure to metabolites, could be related with non-adherence. At all cycles, the mean MARS scores of these six patients were slightly lower compared to the mean MARS scores of the total study population (24.0 vs. 24.7 at cycle 1, 23.7 vs. 24.6 at cycle 3, and 23.3 vs. 24.5 at cycle 5). There were no associations between patients' beliefs (BMQ-subscales or Brief IPQ-subscales) and scores on the MARS-questionnaire.

### Factors associated with dose adjustments

Preceding a dose reduction or any adjustment of the dosing regimen, patients reported several symptoms more frequently as compared to patients who continued their treatment as originally prescribed (Supplementary Table S2). Patients who started cycle 2 with a reduced dose reported more often depression in week 2 of cycle 1 (*p* = 0.025). Skin problems were among the symptoms necessitating any adjustment of the dosing regimen till start of cycle 2 (*p* = 0.004), but these symptoms did not significantly result in dose reduction at start of cycle 2.

The following variables at baseline were positively associated with the occurrence of any adjustment to the dosing regimen during the study period: male (OR 2.89, 95% CI: 1.21–6.91), the number of co-medication (OR 1.16; 95% CI: 1.01–1.31) and five out of eight subscales of the Brief IPQ; consequences (OR 1.24, 95% CI: 1.03–1.48), time line (OR 1.26, 95% CI: 1.04–1.46), identity (OR 1.28, 95% CI: 1.04–1.58), concern (OR 1.19, 95% CI: 1.02–1.40) and emotional response (OR 1.31, 95% CI: 1.11–1.57) (Supplementary Table S3). In multivariate analysis a higher number of co-medication (OR 1.19, 95% CI: 1.03–1.39) and emotional response to the disease (OR 1.32, 95% CI: 1.10–1.59) were associated with the probability of an adjustment of any kind (Supplementary Table S4). Multivariate analyses of factors of the dependent variable “dose reduction at the start of any new cycle” did not result in a significant association.

### Associations between the AUC of metabolites and symptoms

The following relationships between the AUC of capecitabine metabolites and patient-reported symptoms measured during the same cycle were found: AUC 5′-DFUR and weight loss (OR 1.10, 95% CI: 1.01–1.19), AUC FBAL and HFS (OR 0.90, 95% CI: 0.83–0.99), AUC FBAL and rhinorrhea (OR 1.21, 95% CI: 1.03–1.42), AUC FBAL and weight loss (OR 1.09, 95% CI: 1.00–1.20), and AUC FBAL and depression (OR 0.90, 95% CI: 0.82–0.99) (*p* < 0.05) (Supplementary Table S5).

## Discussion

The present study showed that adherence with the use of capecitabine measured with a pill count method was high. Due to the high adherence, factors related to non-adherence were not found. The number of patients reporting non-adherence with MARS, however, increased over time.

Assessing the adherence rate for capecitabine with our pill count method revealed that only 8% of patients used less than 95% and one patient used more than 105% of the prescribed dose. Other studies on capecitabine have also reported high rates of adherence (Macintosh et al., 2007; Mayer et al., 2009; Regnier Denois et al., 2011; Simons et al., 2011; Winterhalder et al., 2011; Bhattacharya et al., 2012; Krolop et al., 2013; Patel et al., 2013; Thivat et al., 2013; Walter et al., 2013; Timmers et al., 2014; Zahrina et al., 2014; De Figueiredo and Forones, 2014). It is not known if the reported non-adherence with capecitabine may result in less efficacy. It should be noted that lowering the initially prescribed dose in the course of treatment in case of toxicity does not seem to compromise clinical efficacy in advanced breast cancer (Cassidy et al., 2002; Leonard et al., 2011), suggesting that some level of non-adherence may not largely affect outcome.

Although, the adherence rate measured with a pill count over the complete study period was high, non-adherence on the basis of the measurement of plasma concentrations was observed. There was an absence of any metabolite in three of 160 samples (1.9%), while the patients had reported to have taken capecitabine in the period 20 min–12 h before blood collection. These patients had detectable metabolite concentrations at other time-points.

Self-reported non-adherence measured with MARS revealed increasing rates of deviations over time from 15% in cycle 1 to 29% in cycle 5. “Forgetting to take pills,” which is considered the main cause of unintentional non-adherence, was the most frequently reported reason (Hugtenburg et al., 2013). Also reports on intentional non-adherence (e.g., adjusting the dose or deciding to skip a dose) increased over time. Experience of adverse events may decrease quality of life, thereby influencing patients' decisions to skip and/or reduce doses. Decreasing adherence over time has been described previously and is especially relevant for longer term oral treatments. One third of patient who discontinued treatment before finishing 5 cycles, stated that side effects were a reason to discontinue. Therefore, measures to support adherence and ameliorate adverse events seem specifically relevant in on-going use and should be incorporated in prolonged support of patients to optimize their use of capecitabine.

The concerns and perceived necessity of medication measured with the BMQ showed that 50% of patients had an accepting attitude toward capecitabine. As compared to patients with asthma, cardiac disease, depression, or diabetes (Tibaldi et al., 2009), more capecitabine patients scored high on the concerns (48 vs. 33%) and necessity (94 vs. 88%) scales. In non-small cell lung cancer patients using erlotinib, we also found high scores for the concerns and necessity scales on the BMQ (Timmers et al., 2015). The seriousness of the disease seems to reflect the differences in BMQ-scores with other patient groups. Although, the relationship between BMQ-scores and adherence has been established thoroughly (Horne et al., 2013), we did not find this relation in our population, probably as a result of the small amount of patients with non-adherence.

Patients experienced a variety of symptoms. HFS, fatigue and gastro-intestinal problems (like flatulence, nausea, and diarrhea), were reported most frequently. As many patients already had symptoms at baseline, were pre-treated and/or used capecitabine in combination with oxaliplatin or irinotecan, it is difficult to differentiate between these factors and adverse events from capecitabine. Since patients report subjective and less specific complaints better than their clinicians (Trotti et al., 2007), ideally patient-reported symptoms should be used complementary to physicians' scores (Basch et al., 2009, 2012). Patient-reported outcomes (PRO) are crucial in patient-centered care, especially in end-of-life care and palliative care where symptom management and quality of life are most important (Peppercorn et al., 2011). PRO to assess the actual benefit patients retrieve from their treatment is increasingly being used in oncology (Gaertner and Becker, 2014). In this respect, it should be noted that the National Cancer Institute recently published a PRO version of the common terminology criteria for adverse events (PRO-CTCAE), which enables the assessment of patient-reported symptoms in cancer clinical research (Basch et al., 2014).

Adjustments in the dosing regimen made by the physician occurred at least once in almost two third of the patients (62%). Most common adjustments were delaying a next cycle and reducing the dose of the next cycle, both occurring in about one third of patients. Patient-reported symptoms were collected in the second week of the cycle, because of which we were not able to analyze the reasons for adjustments after the rest period from the patient's perspective. For physicians, severe adverse events in the preceding treatment cycle as well as the patient's wish or complaints may be reasons to adjust the dose. Depression was more frequently found in the cycle prior to dose reduction. HFS and diarrhea, which are generally physician's reasons to adjust the dose (Walko and Lindley, 2005), were not reported more frequently in patients with dose adjustment. In this respect, we were unable to take into account severe symptoms only, because the number of patients in our study was too small. Emotional response to the disease and the number of co-medication were associated with the occurrence of any adjustment of the dosing regimen during the five treatment cycles. Co-medication is likely related with a poorer health condition with a higher risk of adverse events. A stronger emotional response to cancer may also be a reflection of poorer health condition or may influence a physician's estimation of the level of discomfort caused by adverse events.

We observed few relationships between patient-reported symptoms and the AUC of capecitabine metabolites (AUC 5′-DFUR and weight loss, AUC FBAL and HFS, AUC FBAL and rhinorrhea, AUC FBAL and weight loss, AUC FBAL and depression). None of them have been observed by Gieschke et al. (2003). It is well known that capecitabine clearance is subject to considerable inter-individual variation (Regnier Denois et al., 2011). HFS is a common side-effect of capecitabine which causes serious discomfort to patients and can be the reason for dose adjustment, discontinuation of therapy and may influence medication adherence. Patients on DPD inhibitor-associated fluoropyrimidine treatments, such as uracil/tegafur or S-1, only occasionally experience HFS, which suggests that this adverse event is caused by the metabolites of 5-FU (Yen-Revollo et al., 2008). We, however, found an inverse relationship: a higher AUC of FBAL was associated with a lower risk of HFS (OR 0.90, 95% CI: 0.83–0.99). This inconsistency may be caused by the low incidence of severe HFS in our small population. For 5-FU, therapeutic drug monitoring (TDM) may contribute to reduce toxicity and improve response (Paci et al., 2014), but TDM to optimize dosing of capecitabine does not seem to be useful.

There are strengths and limitations to discuss. Unique is that we longitudinally collected a wide range of variables to explore the use of capecitabine in daily practice. However, the number of patients was limited and some relevant information was not collected (such as number of patients approached and previous or concomitant use of medication). The variety of variables and interrelationships may introduce risk of errors by multiple testing. We did not adjust the significant *p*-value as our intention was to broadly explore daily practice. Future research using specific research questions may confirm the associations. The adherence rate over the studied period was obtained with a pill count method taking into account the dose adjustments made by the physician during treatment. Furthermore, the information was enriched with a self-report method by means of MARS. A disadvantage of these methods is, that they do not provide information on the timing of intake. Although, it was not planned in the design of the study, we identified few blood samples without detectable metabolite concentrations of which it is likely that the patient was non-adherent at that moment. The studied period lasted five cycles. As adherence is decreasing over time, further research should focus on continued use requiring prolonged follow-up. Another limitation of our study is that we did not collect patient-reported symptoms on the day preceding the next cycle, because of which we do not know the reasons for dose adjustments from the patients' perspective. Since the number of patients with an adherence rate <95 or >105% was very low, we were not able to study the influence of attitude or patient-reported symptoms on adherence.

## Conclusion

Adherence to capecitabine was generally high. The clinical relevance of the small extent of non-adherence is uncertain. Medication adherence measured with self-reports (MARS) decreased over time. Side effects were reported by one third of patients as the reason to discontinue treatment. Therefore, it appears that adverse event management is important to support persistence. In addition, adherence management to support implementation of correct capecitabine use is specifically relevant in longer term treatment. With the extending armamentarium of oral targeted anticancer agents and prolonged treatment duration, we expect the issue of medication adherence of increasing importance in oncology.

## Author contributions

LT, JH designed the study, CB, LT coordinated the study, PvdB, AB, and EB included patients, LT, CB and DM collected data, LT, CB, DM, PvdV, ES, RH, GP, EB and JH analyzed data, LT, CB, PvdV, ES, GP, EB, and JH interpreted data, LT wrote the draft version, all contributed to the manuscript writing, all approved the final manuscript.

## Funding

Roche, the Netherlands, has provided an unrestricted grant.

### Conflict of interest statement

The authors declare that the research was conducted in the absence of any commercial or financial relationships that could be construed as a potential conflict of interest.
