# Supplementary material for: Adherence and Patients' Experiences with the Use of Capecitabine in Daily Practice
Source: Front Pharmacol. 2016 Sep 21;7:310. doi: 10.3389/fphar.2016.00310 (PMC5030243; doi:10.3389/fphar.2016.00310)
Supplement: Supplementary file 1 [file DataSheet1.docx]

|  | **Supplementary table S1.** Number of patients for whom data are available at time-point | | | | | | |
| --- | --- | --- | --- | --- | --- | --- | --- |
|  |  |  | T0 | cycle 1 | cycle 3 | cycle 5 |  |
|  | Patients on treatment at start cycle | | 92 | 92 | 77 | 57 |  |
|  | Patients on treatment in week 2 | | n.a. | 87 | 70 | 57 |  |
|  |  |  |  |  |  |  |  |
|  | Adherence rate (AR)^a^ | | n.a. | 67 | 61 | 53 |  |
|  | AUCs blood sample (AUCs)^b^ | | n.a. | 58 | 41 | 38 |  |
|  | Questionnaire (Q)^b^ | | 92 | 77 | 66 | 56 |  |
|  |  |  |  |  |  |  |  |
|  | Patients AR + AUCs | | n.a. | 48 | 36 | 37 |  |
|  | Patients AR + Q | | n.a. | 63 | 59 | 52 |  |
|  | Patients AUCs + Q | | n.a. | 53 | 39 | 37 |  |
|  | Patients AR + AUCs + Q | | n.a. | 46 | 35 | 36 |  |
|  | Abbreviations: AR, Adherence Rate; AUCs, Area Under the Curve of 5’-deoxy-5-fluorouridine | | | | | |  |
|  | (DFUR), 5-fluorouracil (5FU) and α-fluoro-β-alanine (FBAL); Q, Questionnaire; ^a^for patients still | | | | | |  |
|  | on treatment at the start of cycle; ^b^in week 2 of cycle. | | | | | |  |

|  | **Table 2.** Adjustments in dosing regimen made by physician | | | | |  |  |  |
| --- | --- | --- | --- | --- | --- | --- | --- | --- |
|  |  | Cycle 1 | Cycle 2 | Cycle 3 | Cycle 4 | Cycle 5 | Cycle 1-5 |  |
|  |  | n=92 | n=85 | n=77 | n=62 | n=57 | n=92 |  |
|  | Dose at start of cycle |  |  |  |  |  |  |  |
|  | median (mg/m^2^/d) | 1975 | 1970 | 1977 | 1977 | 1950 |  |  |
|  | range (mg/m^2^/d) | 1046 - 2609 | 1141 - 2609 | 1210 - 2556 | 1068 - 2378 | 1158 - 2290 |  |  |
|  | median (mg/d) | 4000 | 4000 | 36000 | 36000 | 3600 |  |  |
|  | range (mg/d) | 2000 - 5300 | 1000 - 5300 | 1000 - 5000 | 2000 - 5000 | 2000 - 5000 |  |  |
|  | Dose adjustments |  |  |  |  |  |  |  |
|  | at start of cycle^a^ (% patients) | n.a. | 17.6 | 7.8 | 17.7 | 7.0 | 41.3 |  |
|  | - dose reduction^a^ (% patients) | n.a. | 12.9 | 6.5 | 17.7 | 5.3 | 30.4 |  |
|  | - dose increase^a^ (% patients) | n.a. | 4.7 | 1.3 | 0 | 1.8 | 13.0 |  |
|  | during cycle^b^ (% patients) | 4.3 | 3.6 | 2.6 | 0 | 0 | 7.6 |  |
|  | - dose reduction^b^ (% patients) | 4.3 | 2.4 | 2.6 | 0 | 0 | 7.6 |  |
|  | - dose increase^b^ (% patients) | 0 | 1.2 | 0 | 0 | 0 | 1.1 |  |
|  | Shortening period of use | 7.6 | 4.7 | 10.4 | 4.8 | 1.8 | 20.7 |  |
|  | # days shortened (median) | 1 | 1 | 3 | 4 | 8 |  |  |
|  | # days shortened (range) | 1-6 | 1-12 | 1-8 | 1-6 | 8-8 |  |  |
|  | Cycle delay (% patients)* | 7.6 | 12.9 | 8.0 | 19.4 | 17.5 | 34.8 |  |
|  | # days extended (median) | 7 | 8 | 11 | 11 | 7 |  |  |
|  | # days extended (range) | 2-12 | 1-20 | 2-11 | 2-21 | 1-7 |  |  |
|  | Any adjustment in dosing regimen | 15.2 | 32.9 | 28.6 | 32.3 | 22.8 | 62.0 |  |
|  | Abbreviations: sd, standard deviation; mg/m2/d, milligrams per square meters per day; mg/d, milligrams per day. | | | | | |  |  |
|  | ^a^adjustment at start cycle compared to dose at start of previous cycle. ^b^:temporarely adjustment during cycle. | | | | | |  |  |
|  | * Patients who did not start the next cycle were not classified as with delay. | | | |  |  |  |  |

| **Supplementary table S3.** Symptoms at cycle 1 preceding dose reduction or any | | | | | | |
| --- | --- | --- | --- | --- | --- | --- |
| adjustment to the dosing regimen | | | | | | |
|  | Dose reduction at start cycle 2 | | | Any adjustment to dosing regimen till start cycle 2* | | |
|  | yes N=8^a^ | no N=67^a^ | *p*-value | yes N=20^a^ | no N=56^a^ | *p*-value |
| Any symptom at cycle 1 | % | % |  | % | % |  |
| Hand-foot syndrome | 67 | 67 | 1.000 | 80.0 | 63.6 | 0.351 |
| Fatigue | 100 | 82 | 0.581 | 100.0 | 78.6 | **0.032** |
| Diarrhea | 50 | 38 | 0.670 | 47.1 | 37.0 | 0.461 |
| Nausea | 83 | 44 | 0.097 | 76.5 | 39.6 | **0.008** |
| Stomach Ache | 33 | 32 | 1.000 | 35.3 | 32.1 | 0.809 |
| Flatulence | 83 | 59 | 0.392 | 70.6 | 58.2 | 0.359 |
| Constipation | 67 | 34 | 0.182 | 64.7 | 29.1 | **0.008** |
| Mucositis | 0 | 14 | 1.000 | 23.5 | 10.9 | 0.232 |
| Skin problems | 50 | 26 | 0.338 | 55.6 | 20.0 | **0.004** |
| Alopecia | 0 | 11 | 1.000 | 17.6 | 8.9 | 0.378 |
| Nail problems | 17 | 8 | 0.423 | 11.8 | 7.3 | 0.621 |
| Dry mouth | 67 | 42 | 0.393 | 76.5 | 34.5 | **0.002** |
| Breathing problems | 50 | 37 | 0.669 | 38.9 | 39.3 | 0.976 |
| Rhinorrhea | 50 | 30 | 0.376 | 41.2 | 30.4 | 0.406 |
| Muscular pain | 67 | 38 | 0.212 | 50.0 | 38.2 | 0.376 |
| Eye problems | 17 | 17 | 1.000 | 17.6 | 17.9 | 1.000 |
| Headache | 67 | 30 | 0.086 | 47.1 | 29.6 | 0.185 |
| Dizziness | 50 | 29 | 0.365 | 47.1 | 27.3 | 0.126 |
| Change of taste | 67 | 50 | 0.736 | 66.7 | 47.3 | 0.153 |
| Loss of appetite | 67 | 46 | 0.107 | 88.9 | 37.5 | **<0.001** |
| Weight loss | 33 | 46 | 0.686 | 52.9 | 42.9 | 0.464 |
| Fever | 17 | 14 | 1.000 | 23.5 | 12.7 | 0.275 |
| Oedema | 0 | 8 | 1.000 | 11.8 | 7.3 | 0.621 |
| Insomnia | 50 | 29 | 0.361 | 41.2 | 28.6 | 0.327 |
| Depression | 83 | 33 | **0.025** | 70.6 | 28.6 | **0.002** |
| Abbreviations: any, any grade of symptom. * Shortening of period of use of cycle 1, delaying | | | | | | |
| cycle 2 and/or decreasing the starting dose of cycle 2. ^a^Missings excluded in frequency | | | | | | |
| analyses. | | | | | | |

|  | **Supplementary Table S4.** Univariate and multivariate analyses on any adjustment of | | | | | | | |  |
| --- | --- | --- | --- | --- | --- | --- | --- | --- | --- |
|  | original dosing regimen made by the physician | | |  |  |  |  |  |  |
|  |  | Univariate analysis | | |  | Multivariate analysis | | |  |
|  |  | OR | 95% CI | *p*-value |  | OR | 95% CI | *p*-value |  |
|  | Sex | 2.89 | 1.21-6.91 | 0.017 | * |  |  |  |  |
|  | Age | 1.02 | 0.97-1.07 | 0.479 |  |  |  |  |  |
|  | Education | 0.87 | 0.34-2.22 | 0.871 |  |  |  |  |  |
|  | Living status | 0.86 | 0.29-2.55 | 0.782 |  |  |  |  |  |
|  | Occupation | 0.43 | 0.16-1.17 | 0.097 |  |  |  |  |  |
|  | Number of co-medication | 1.16 | 1.01-1.33 | 0.033 | * | 1.19 | 1.03 - 1.39 | 0.020 | * |
|  | SF-12 |  |  |  |  |  |  |  |  |
|  | Physical component | 0.97 | 0.92-1.01 | 0.163 |  |  |  |  |  |
|  | Mental component | 0.98 | 0.92-1.03 | 0.382 |  |  |  |  |  |
|  | BMQ |  |  |  |  |  |  |  |  |
|  | General overuse | 1.00 | 0.84-1.19 | 0.972 |  |  |  |  |  |
|  | General harm | 1.09 | 0.89-1.33 | 0.418 |  |  |  |  |  |
|  | Specific necessity | 1.11 | 0.97-1.27 | 0.142 |  |  |  |  |  |
|  | Specific concern | 1.12 | 0.996-1.26 | 0.058 |  |  |  |  |  |
|  | Nec-conc differential | 0.98 | 0.90-1.07 | 0.632 |  |  |  |  |  |
|  | Brief IPQ |  |  |  |  |  |  |  |  |
|  | Consequences | 1.24 | 1.03-1.48 | 0.022 | * |  |  |  |  |
|  | Time line | 1.26 | 1.04-1.46 | 0.015 | * |  |  |  |  |
|  | Personal control | 1.04 | 0.90-1.20 | 0.632 |  |  |  |  |  |
|  | Treatment control | 1.05 | 0.83-1.32 | 0.713 |  |  |  |  |  |
|  | Identity | 1.28 | 1.04-1.58 | 0.019 | * |  |  |  |  |
|  | Concern | 1.19 | 1.02-1.40 | 0.032 | * |  |  |  |  |
|  | Understanding | 0.98 | 0.85-1.13 | 0.749 |  |  |  |  |  |
|  | Emotional response | 1.31 | 1.10-1.57 | 0.002 | * | 1.32 | 1.10-1.59 | 0.003 | * |
|  | Abbreviations: CI , confidence interval, SF-12, SF-12 Health Survey; BMQ, Beliefs about Medicines | | | | | | | |  |
|  | Scale; Nec-conc, Necessity-concerns; Brief IPQ, Brief Illness Perception Questionnaire; 5'-DFUR, | | | | | | | |  |
|  | 5’-deoxy-5-fluorouridine; 5-FU, '5-fluorouracil; FBAL, 'α-fluoro-β-alanine. *significance (p<0.05) | | | | | | | |  |

|  | **Supplementary table S5.** Relationship between AUC of 5'-DFUR, 5-FU, FBAL and symptom | | | | | | | | |
| --- | --- | --- | --- | --- | --- | --- | --- | --- | --- |
|  |  |  | **5'-DFUR** | | **5-FU** | | **FBAL** | |  |
|  | Any symptom | cases | OR | 95%CI | OR | 95%CI | OR | 95%CI |  |
|  | Hand-foot syndrome | 76 | 0.93 | [0.86-1.01] | 0.88 | [0.51-1.53] | **0.90*** | **[0.83-0.99]** |  |
|  | Fatigue | 76 | 1.02 | [0.95-1.10] | 1.66 | [0.95-2.92] | 1.06 | [0.96-1.17] |  |
|  | Diarrhea | 73 | 1.05 | [0.97-1.13] | 0.52 | [0.25-1.06] | 1.01 | [0.95-1.08] |  |
|  | Nausea | 75 | 1.03 | [0.97-1.10] | 0.83 | [0.56-1.22] | 0.99 | [0.97-1.02] |  |
|  | Stomach ache | 76 | 1.03 | [0.92-1.15] | 1.77 | [0.61-5.16] | 1.03 | [0.93-1.15] |  |
|  | Flatulance | 73 | 0.98 | [0.89-1.07] | 0.94 | [0.34-2.59] | 0.99 | [0.91-1.07] |  |
|  | Obstipation | 75 | 1.05 | [0.95-1.16] | 0.57 | [0.23-1.39] | 1.00 | [0.93-1.09] |  |
|  | Mucositis | 75 | 1.03 | [0.93-1.14] | 0.60 | [0.29-1.08] | 1.02 | [0.93-1.11] |  |
|  | Skin problems | 76 | 0.99 | [0.90-1.09] | 0.60 | [0.26-1.41] | 0.93 | [0.84-1.02] |  |
|  | Alopecia | 74 | 1.02 | [0.96-1.08] | 1.21 | [0.74-1.97] | 1.02 | [0.96-1.08] |  |
|  | Nail problems | 74 | 1.02 | [0.94-1.11] | 0.77 | [0.40-1.51] | 0.97 | [0.89-1.05] |  |
|  | Dry mouth | 76 | 0.94 | [0.85-1.05] | 0.83 | [0.43-1.62] | 0.91 | [0.82-1.01] |  |
|  | Breathing problems | 76 | 0.96 | [0.86-1.06] | 1.30 | [0.61-2.77] | 0.95 | [0.87-1.04] |  |
|  | Rhinorrhea | 75 | 1.08 | [0.95-1.22] | 1.72 | [0.61-4.83] | **1.21*** | **[1.03-1.42]** |  |
|  | Muscular pain | 75 | 1.10 | [0.98-1.24] | 0.50 | [0.20-1.28] | 1.00 | [0.92-1.09] |  |
|  | Eye problems | 74 | 0.99 | [0.92-1.07] | 1.07 | [0.81-1.41] | 1.00 | [0.93-1.08] |  |
|  | Headache | 75 | 1.04 | [0.99-1.09] | 0.96 | [0.57-1.59] | 1.03 | [0.97-1.08] |  |
|  | Dizziness | 75 | 1.02 | [0.93-1.12] | 1.04 | [0.47-2.29] | 1.03 | [0.92-1.14] |  |
|  | Change of taste | 76 | 1.02 | [0.96-1.09] | 1.28 | [0.60-2.71] | 1.04 | [0.97-1.12] |  |
|  | Loss of appetite | 74 | 0.99 | [0.91-1.08] | 1.02 | [0.54-1.93] | 1.01 | [0.92-1.10] |  |
|  | Weight loss | 74 | **1.10*** | **[1.01-1.19]** | 0.83 | [0.43-1.58] | **1.09*** | **[1.00-1.20]** |  |
|  | Fever | 76 | 1.00 | [0.90-1.11] | 0.93 | [0.46-1.90] | 1.02 | [0.93-1.12] |  |
|  | Oedema | 76 | 1.06 | [0.99-1.14] | 1.01 | [0.79-1.28] | 1.07 | [0.99-1.15] |  |
|  | Insomnia | 76 | 0.99 | [0.93-1.04] | 0.81 | [0.41-1.61] | 0.95 | [0.89-1.02] |  |
|  | Depression | 76 | 0.94 | [0.87-1.01] | 0.85 | [0.39-1.85] | **0.90*** | **[0.82-0.99]** |  |
|  | Abbreviations: 5’-DFUR, 5’-deoxy-5'-fluorouridine; 5-FU, 5-fluorouracil; FBAL, α-fluoro-β-alanine; | | | | | | | |  |
|  | AUC, area under the curve; OR, odds ratio; CI,95% confidence interval. *significance (p<0.05) | | | | | | | |  |
|  | NB: calculated with Generalized Estimating Equations for all cases in cycles 3 and 5. | | | | | | | |  |
